# Characterization of the hoof bacterial communities in feedlot cattle affected with digital dermatitis, foot rot or both using a surface swab technique

**DOI:** 10.1186/s42523-023-00277-1

**Published:** 2024-01-22

**Authors:** Nicholas S. T. Wong, Nilusha Malmuthge, Désirée Gellatly, Wiolene M. Nordi, Trevor W. Alexander, Rodrigo Ortega Polo, Eugene Janzen, Karen Schwartzkopf-Genswein, Murray Jelinski

**Affiliations:** 1 Western College of Veterinary Medicine, University of Saskatchewan, Saskatoon, Canada; 2 Lethbridge Research and Development Centre, Agriculture and Agri-Food Canada, Lethbridge, Canada; 3 Technology Access Centre for Livestock, Olds College of Agriculture and Technology, Olds, Canada; 4 Faculty of Veterinary Medicine, University of Calgary, Calgary, Canada

**Keywords:** Hoof lesions, 16S amplicon sequencing, Beef cattle, Lameness, Swab

## Abstract

**Background:**

Lameness is defined as altered or abnormal gait due to dysfunction of the locomotor system, and is a health issue of feedlot cattle, having major economic, labour, and welfare implications. Digital dermatitis (DD—a lesion of the plantar surface of the foot) and foot rot (FR—affects the interdigital cleft) are common infectious causes of lameness in feedlots. These hoof lesions can occur alone or in combination (DD + FR) in the same hoof. A total of 208 hoof swabs were collected from three commercial feedlots located in southern Alberta. Every lesion sample was matched with a corresponding control skin sample taken from a healthy contralateral foot. Control skin samples were also collected from cattle with no lesion on any feet. Bacterial communities of three types of hoof lesions (DD, DD + FR, FR) and healthy skin were profiled using 16S amplicon sequencing.

**Results:**

Alpha diversity analysis revealed a lower bacterial diversity on DD and FR lesions compared to control skin. Beta diversity analysis showed that bacterial communities of DD, FR, and DD + FR lesions were distinct from those of the control skin. While the impact of feedlot was minimal, lesion type contributed to 22% of the variation observed among bacterial communities (PERMANOVA-R = 0.22, *P *< 0.01). Compared to the corresponding control skin, there were 11, 12, and 3 differentially abundant (DA) bacterial genera in DD, DD + FR, and FR lesions, respectively.

**Conclusions:**

The bacterial community description of a DD + FR lesion is a novel finding. Not only did lesions lead to altered bacterial communities when compared to healthy skin, but the composition of those communities also differed depending on the hoof lesion. The 16S amplicon sequencing of surface swabs has significant value as a research tool in separating different hoof lesions and can provide additional insights to the polybacterial etiology of DD and FR in feedlot cattle.

**Supplementary Information:**

The online version contains supplementary material available at 10.1186/s42523-023-00277-1.

## Background

Cattle lameness is a significant production and animal welfare issue in feedlots. Economically, the cost per lameness case is estimated to be between $122–$1,391 CAD [1]. Besides the logistical challenges related to identifying, diagnosing, and treating lame cattle, lameness is associated with acute and chronic pain in cattle with undesirable animal welfare outcomes [2]. Most lameness cases can be attributed to infections of the hoof [3]. A recent Alberta feedlot study reported that lameness accounted for 30.3 to 42.2% of all treated cattle across two years, with interdigital necrobacillosis, also known as interdigital phlegmon (IP) or foot rot (FR), being the most commonly treated lameness, followed by digital dermatitis (DD) [4]. FR affects the interdigital space and is the most common cause of hoof related lameness in feedlot cattle. The interdigital tissue may subsequently become necrotic, leading to a characteristic foul odour. Based on early bacteriology studies, *Fusobacterium necrophorum*, *Prevotella melaninogenicus, Porphyromonas levii,* and *Dichelobacter nodosus* were considered the primary etiological agents of FR [5–7]. However, recent metagenomic studies have identified additional FR-associated bacteria in dairy cattle [8, 9]. Systemic antimicrobials are generally effective treatments for FR [10], thus it is the main treatment option.

Digital dermatitis (DD), also known as papillomatous digital dermatitis, hairy heel warts, and strawberry foot rot [10], is ubiquitous in dairy industries worldwide and  has become emergent in beef cattle during the past decade [4, 11]. DD lesions are primarily found on the plantar regions of the hoof around the dew claws, which can lead to moderate or severe lameness. DD lesions can develop non-linearly through a series of four main distinct morphological stages. Thus, DD lesions range from early lesion (M1) to active lesions (M2, M4.1), but can also become chronic (M4) or become healed and scabbed (M3) [12, 13]. *Treponema* species are consistently found in DD lesions of both beef and dairy cattle and are considered the main causative agent [14, 15]. A recent study of DD lesion microbial communities showed that *Treponema, Mycoplasma, Porphyromonas,* and *Fusobacterium* were core genera that distinguished the DD lesion from that of normal skin [16]. DD treatments typically involve the application of a topical treatment such as oxytetracycline spray, salicylic acid, and foot bathing, but research has shown mixed efficacies [17, 18].

Although DD and FR primarily affect different skin areas of the hoof, both lesions can simultaneously occur within the same hoof (DD + FR). In addition to lameness being the common clinical sign for these two distinct hoof lesions, their clinical presentations may be similar. Thus, accurate diagnosis is contingent upon a knowledgeable observer performing a thorough physical examination. Distinguishing these two types of hoof lesions is especially important because each has different treatment protocols and prognoses. Cattle with FR generally respond well with parenteral antibiotic injection, but successful DD treatment is dependent on topical application of an antimicrobial, which can be a more laborious process. Subsequent monitoring of treated DD lesions may also be needed should DD lesions recur. Because of the relative complexity for treating DD, accurate determination of the nature of hoof lesion is crucial. Having an objective method, such as microbial testing via a swab, to distinguish between lesion types can improve issues associated with misdiagnosing, which can be especially difficult in a feedlot environment.

16S amplicon sequencing is a popular culture-independent method used to profile host-associated bacterial communities and has been used to study bacterial communities of hoof lesions [19]. Despite FR being the most prevalent cause of lameness in feedlot cattle [20], FR bacterial communities have not been extensively researched. Furthermore, FR research has been based on culture-dependent techniques and to date, only one study describes the lesion community of FR in dairy cattle [8]. In comparison to FR, there has been greater interest in conducting microbiome studies on DD [19]. However, these DD studies have been mostly limited to dairy cattle and utilized skin biopsy as a method to sample bacterial communities. While lameness is a major concern of the beef industry, invasive sampling is not a practical option to evaluate the hoof microbial communities of beef cattle with lesions. Compared to punch biopsies, surface swabs are less invasive and a more practical procedure to collect samples in feedlot operations. Past studies reported that *Treponema, Fusobacterium, Porphyromonas,* and *Dichelobacter* are bacterial genera associated with both DD and FR [9, 16, 21]. This study’s aim was to characterize and compare the lesion or control skin surface bacterial communities of feedlot cattle affected with DD, FR, or DD + FR using surface swab samples. It is hypothesized that lesion surface communities would have a different bacterial composition compared to surface communities of healthy skin tissues. *Fusobacterium* and *Treponema* are classic hoof pathogens expected to be in high abundance in FR and DD lesions, respectively; DD + FR combination lesion communities are predicted to contain biomarkers from both types of lesions.

## Methods

### Sample collection

**Fig. 1:**
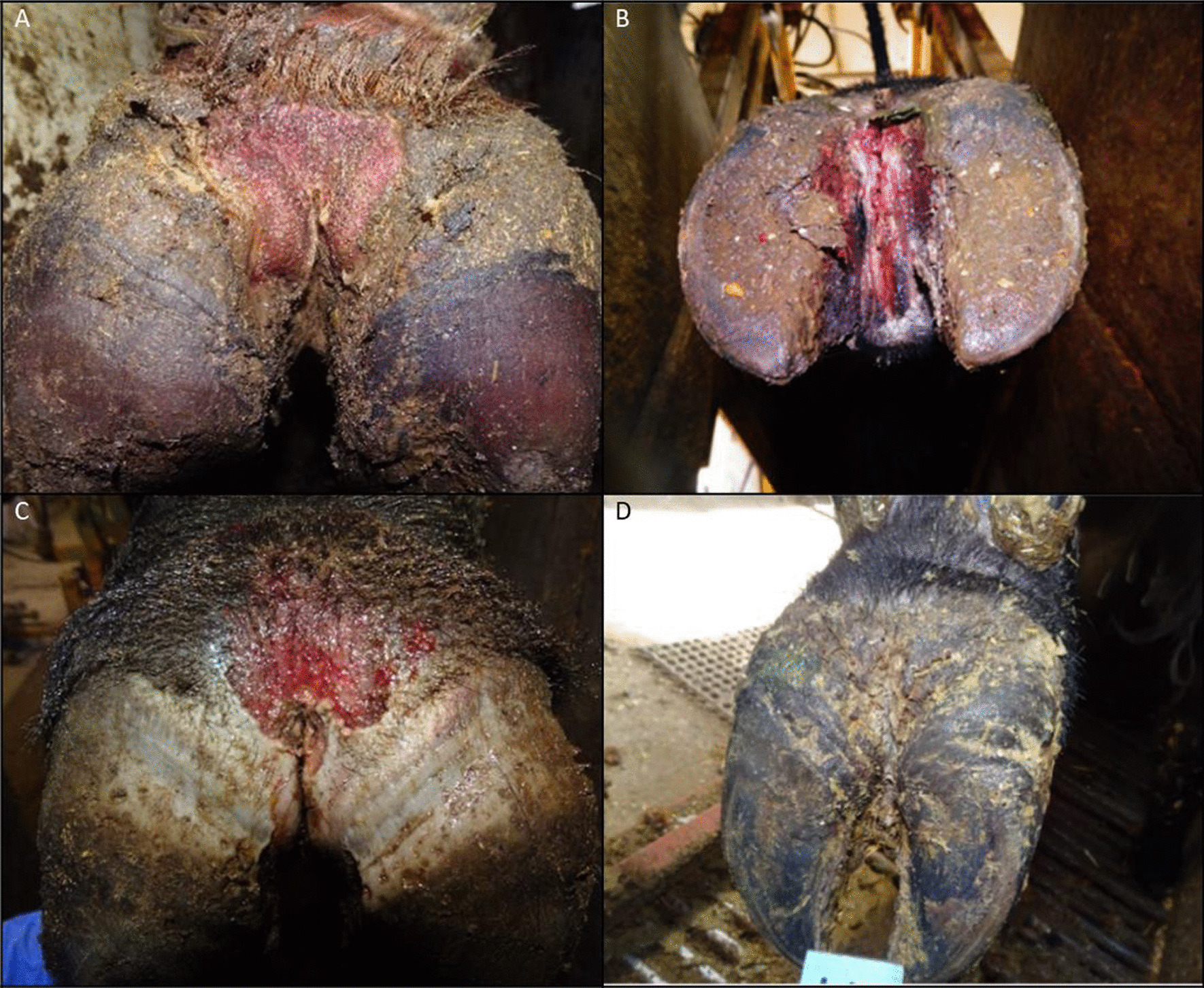
**A** A foot with an M2 stage DD lesion near the heel bulb region **B** A foot with a FR lesion in the interdigital space with notable swelling above the coronary band causing the claws to splay. **C** A splayed foot with both an M2 stage DD lesion near the heel bulb region, and a FR lesion in the interdigital cleft. **D** A healthy control foot. DD = Digital dermatitis, FR = Foot rot, DD + FR = DD and FR co-infection in the same foot

From January 2019 to March 2020, weekly visits were made to one of three commercial feedlots (> 20,000 head capacity) in southern Alberta. A total of 101 animals of all three feedlots were housed in large outdoor dirt pens, consisting of Angus, Charolais, Herefords, Short horn, Simmental, Speckled Park, and Crossbred cattle. See Additional file 1: Table S1 for biological information summary of the sampled cattle from each feedlot. Researchers from the Agriculture and Agri-Food Canada Lethbridge Research and Development Centre (LRDC, Lethbridge, AB, Canada), were trained and experienced in conducting visual assessments of hoof lesions on lame cattle to determine the cause. On the morning of each visit, feedlot staff placed cattle exhibiting signs of lameness into a holding pen next to a handling facility. Animals were then moved calmly by one handler and scored by an experienced observer according to the level of lameness using the Step Up® beef locomotion scoring system (Zinpro, USA): 0—normal gait. 1—initial signs of lameness, no obvious sign of limp. 2—obvious limp, slight head bob, and arched back. 3—animal applies little to no weight on the affected limb and may be reluctant and unable to move. Each lame animal was then moved to the handling facility and restrained in a squeeze chute, where its affected hoof was examined for evidence of DD, FR, and DD + FR (Fig. 1).

Lesion samples were collected by rotating a sterile cotton-tipped applicator (Puritan Medical Products company, USA) 360 degrees on the surface of the lesion. DD lesion samples were typically collected from the plantar aspect of the hoof, while FR lesion samples were collected from the interdigital cleft. In DD + FR, a single swab was used to collect surface lesion materials from the center of the lesions. For every lesion sample swabbed (DD lesion, FR lesion, and DD + FR lesion), a corresponding control skin sample from the same animal was collected from the same skin region of the contralateral hoof. Hence, DD control is the corresponding control hoof sample opposite to a hoof with DD lesion(s); DD + FR control is the corresponding control hoof sample opposite to a hoof with DD + FR lesion(s); FR control is the corresponding control hoof sample opposite to a hoof with FR lesion(s). Additionally, CH control samples were taken from the interdigital and plantar skin regions of completely healthy cattle that were free of any hoof lesions. These hoof swabs were immediately placed into sterile tubes and placed on ice until storage at − 80 °C.

### DNA extraction

Genomic DNA was extracted from the cotton swabs using the Qiagen DNeasy Powerlyzer/Powersoil kit (Qiagen, USA) according to the manufacturer’s instructions. DNA concentration was measured using the Qubit dsDNA Quantification HS (High Sensitivity) Assay kit (ThermoFisher Scientific, USA), and quality was checked using 1% agarose gel electrophoresis. Extracted samples were stored at − 20 °C. Samples were diluted to 10 ng/µl and transferred onto 96-well plates to prepare amplicons for sequencing. For samples below the 10 ng/µl concentration threshold, a maximum of 10 µl volume was used to prepare amplicons.

### 16S rRNA amplicon sequencing

The sequencing library preparation and sequencing were performed by Genome Quebec (Montreal, Quebec, Canada). The V4 hypervariable region of the 16S rRNA gene was amplified with the primer set 515F (5′–ACACTGACGACATGGTTCTACAGTGCCAGCMGCCGCGGTAA-3′)/806R (3′–TACGGTAGCAGAGACTTGGTCTGGACTACHVGGGTWTCTAAT-5′) [22]. Briefly, the PCR amplification was performed using the Roche FastStart master mix under the following conditions: 94 °C for 2 min, followed by 26 cycles of 94 °C for 30 s, 58 °C for 30 s, 72 °C for 7 min, and a final elongation step at 72 °C for 7 min (Roche Applied Science, Germany). Barcoding was done by using an in-house PCR pipeline at Genome Quebec. Verification of barcode incorporation for each sample was performed on 2% agarose gel. Quantification of each amplicon was conducted with the Quant-iT™ PicoGreen® dsDNA Assay Kit (Life Technologies, USA). A sequencing library was generated by pooling the same quantity (ng) of each amplicon. Following clean up, the sequencing library was quantified using Kapa Illumina GA with the Revised Primers-SYBR Fast Universal kit (Kapa Biosystems Inc, USA) and average fragment size was determined using a LabChip GX instrument (PerkinElmer Inc., USA). Sequencing was performed with the MiSeq Reagent Kit v2 500 cycles from Illumina (2 × 250 bp) with LNATM modified custom primers (Illumina Inc., USA). The LNATN modified custom primers were: Primer read1: LNA-CS1: ACACTGACGACATGGTTCTACA, Primer read2: LNA-CS2: TACGGTAGCAGAGACTTGGTCT, Primer index read: LNA-CS2rc: AGACCAAGTCTCTGCTACCGTA.

### Quality control and sequence data analysis

Raw FASTQ files were downloaded and imported to QIIME2 version 2021.4 [23] in paired end FASTQ Phred 33 format. De-noising was performed using DADA2 in QIIME2 to merge paired reads, filter and denoise sequences, and remove chimeric sequences. Based on the Phred score of the sequences, the truncation length was set at 240 base pairs for both the forward and reverse reads [24]. Representative sequences and an amplicon sequence variants (ASV) tables were generated after the original data was filtered through the DADA2 pipeline. The align-to-tree-mafft-fasttree plug-in was used to align representative sequences and to construct a phylogenetic tree. A feature classifier, classify-sklearn, was used to assign taxonomic ranks using the Silva database version 138 classifier trained for the V4 hypervariable region of the 16S rRNA gene. Filter featuring was performed to remove mitochondria, chloroplasts, and archaea from the ASV table. In addition, unassigned ASVs (eg., taxonomic assignment only at Kingdom: Bacteria, Kingdom: Unassigned) were removed using a feature filtering option. Only taxa at level 6 (genus level) that appeared in more than 10% of all samples and appeared in more than 10% of total frequency were kept for diversity and differential abundance analysis.

### Diversity analyses

The alpha-rarefaction plug-in was used to generate rarefaction curves for each individual sample, using minimum sampling depth [23]. The core-metric-phylogenetic plugin generated alpha and beta diversity metrics. The Shannon and Chao1 indices were included as alpha diversity measures to assess richness and evenness within bacterial communities generated from various lesions and control skin samples. The alpha-group-significance plugin was used to perform a Kruskal–Wallis, non-parametric test with a false-discovery rate (FDR) correction or a Benjamini–Hochberg adjusted *p* value [25].

Beta diversity analysis was performed using unweighted and weighted Unifrac distance metrices, and principal coordinates analysis (PCoA) plots were generated using the Emperor tool to visualize the dissimilarity between bacterial communities [23]. The beta-group-significance and adonis plugin were used to assess the significant differences between bacterial communities using a permutational multivariate analysis of variance (PERMANOVA) non-parametric test [26]. *Post-hoc* pairwise comparisons were performed between a lesion type and its corresponding control skin. Adjusted *p* values were generated using the Benjamini–Hochberg procedure (1995) [25].

### Differential abundance analysis

The ALDEx2 q2-plugin was used to assess differentially abundant (DA) bacterial taxa between the lesion and control skin at level 6 [27]. Three pairwise analyses were performed separately for each lesion type (DD, FR, DD + FR) against the respective healthy control skin. Within the ALDEx2 analysis, centered log-ratios (CLR) of bacterial taxa and Welch’s t-test were used to identify DA bacterial taxa. Adjusted *p* values were calculated using Benjamini–Hochberg procedure, and DA taxa were declared at adjusted *p* value ≤ 0.05 and effect size ≥ 0.8. A positive effect size signified that a taxon was more abundant in lesion when compared to control skin, whereas a negative effect size signified a taxon was more abundant in control compared to lesion skin.

The Songbird q2-plug-in was used to explore links between bacterial taxa and lesion types at level 6 [28]. It utilizes a multinomial regression approach to provide information on the relative association of bacteria with a given covariate. Instead of ALDEx2’s pairwise comparison approach for each lesion-type and its corresponding control, the entire dataset was included in the same model, including CH control. The parameters used in the Songbird analysis included the metadata columns final category (sample type) and feedlot. Thus, the feedlot of origin can be accounted for in the model comparing taxa between final categories (sample types). When the final category + feedlot model was compared to a “null” model, a positive pseudo-Q^2^ value indicated that the model was not over-fitted. Lesion-specific taxa were identified by sorting from the highest to lowest taxa associated with each lesion type in the songbird output.

### Taxonomic summary

The taxa barplot plug-in generated taxonomic bar plots on a filtered table collapsed at the phylum level (level 2). These bar plots show the relative abundance (%) of the 10 most prevalent phyla across all samples. Each bar represents a hoof lesion or control category.

## Results

### Sequence statistics

DADA2 processed 3,221,804 sequences from 208 samples into 29,966 ASVs, with the median frequency of 15,768 reads per sample. These 208 samples consisted of DD lesion (n = 23), DD control (n = 16), DD + FR lesion (n = 25), DD + FR control (n = 22), FR lesion (n = 62), FR control (n = 54), and CH control (n = 6). Refer to Table 1 for distribution of samples across feedlots. Separately, three extraction blank samples each had 354 or fewer sequences, which were significantly less than the hoof sample with the lowest number of sequences (3821). These extraction blank samples were not included in the 208 samples and in the downstream analysis. See Additional file 2: Table S2 for quality control test results.

**Table 1:** Distribution of samples across three feedlots by hoof lesion type and control skin categories

	DD lesion	DD control	DD + FR lesion	DD + FR control	FR lesion	FR control	CH control
Feedlot A	5	3	25	22	51	45	6
Feedlot B	16	11	0	0	0	0	0
Feedlot C	2	2	0	0	11	9	0
Total	23	16	25	22	62	54	6

CH control, DD + FR lesion and DD + FR control samples are from feedlot A only

Feedlot B contributed DD lesion and DD control samples only

*DD* Digital dermatitis, *FR* Foot rot, *DD* + *FR* DD and FR co-infection in the same foot, *CH control* Control skin from completely healthy animals without any hoof lesions

### Diversity analyses

**Fig. 2:**
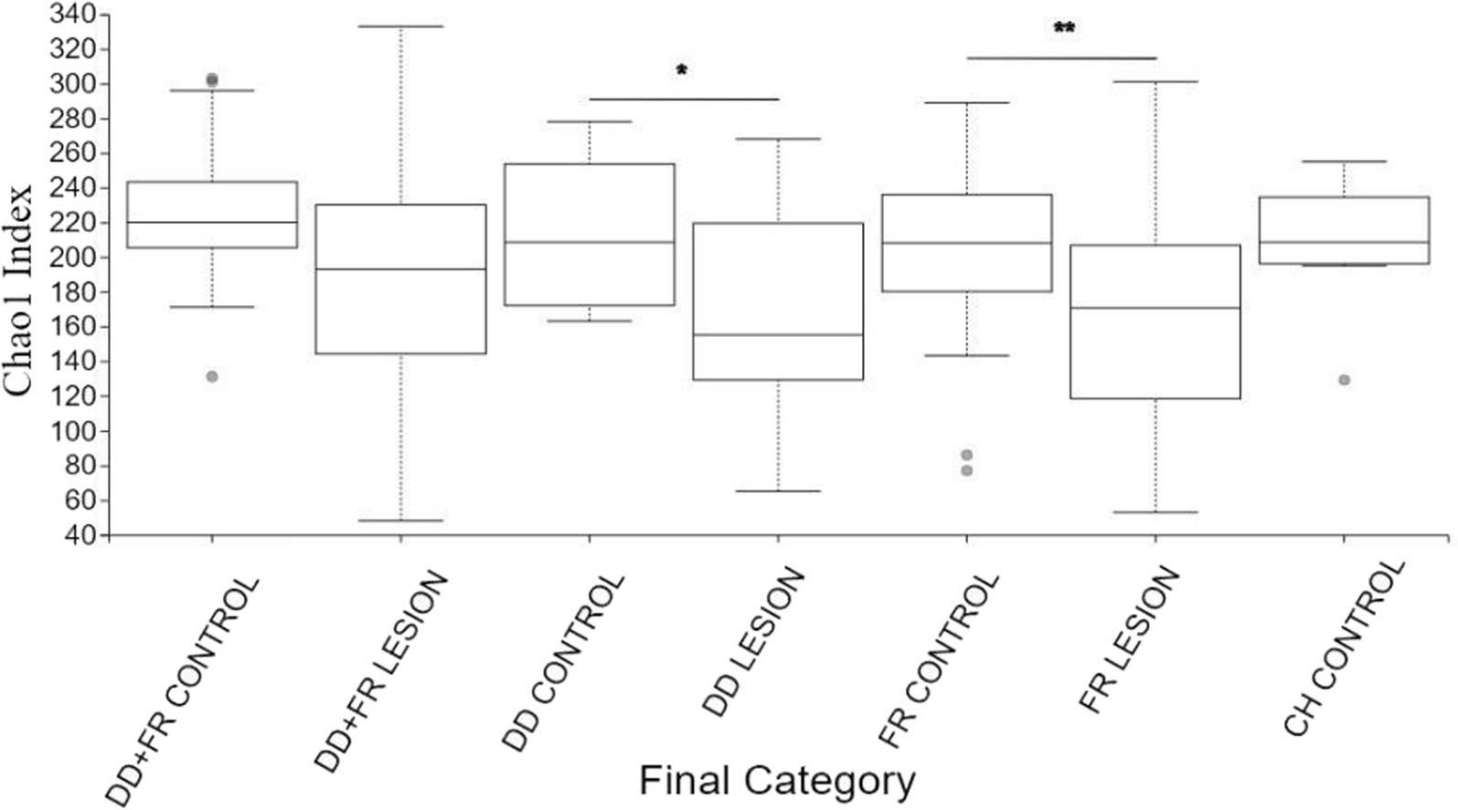
Chao1 diversity index of bacterial communities of DD + FR control, DD + FR lesion, DD control, DD lesion, FR control, FR lesion and CH control. Pairwise analysis compares Chao1 index of lesion and control skin microbial communities within a lesion type. *indicates an adjusted *p* value between 0.01 and 0.05, **indicates an adjusted *p* value < 0.01. DD = Digital dermatitis, FR = Foot rot, DD + FR = DD and FR co-infection in the same foot, CH control = control skin from completely healthy animals without any hoof lesions

**Fig. 3:**
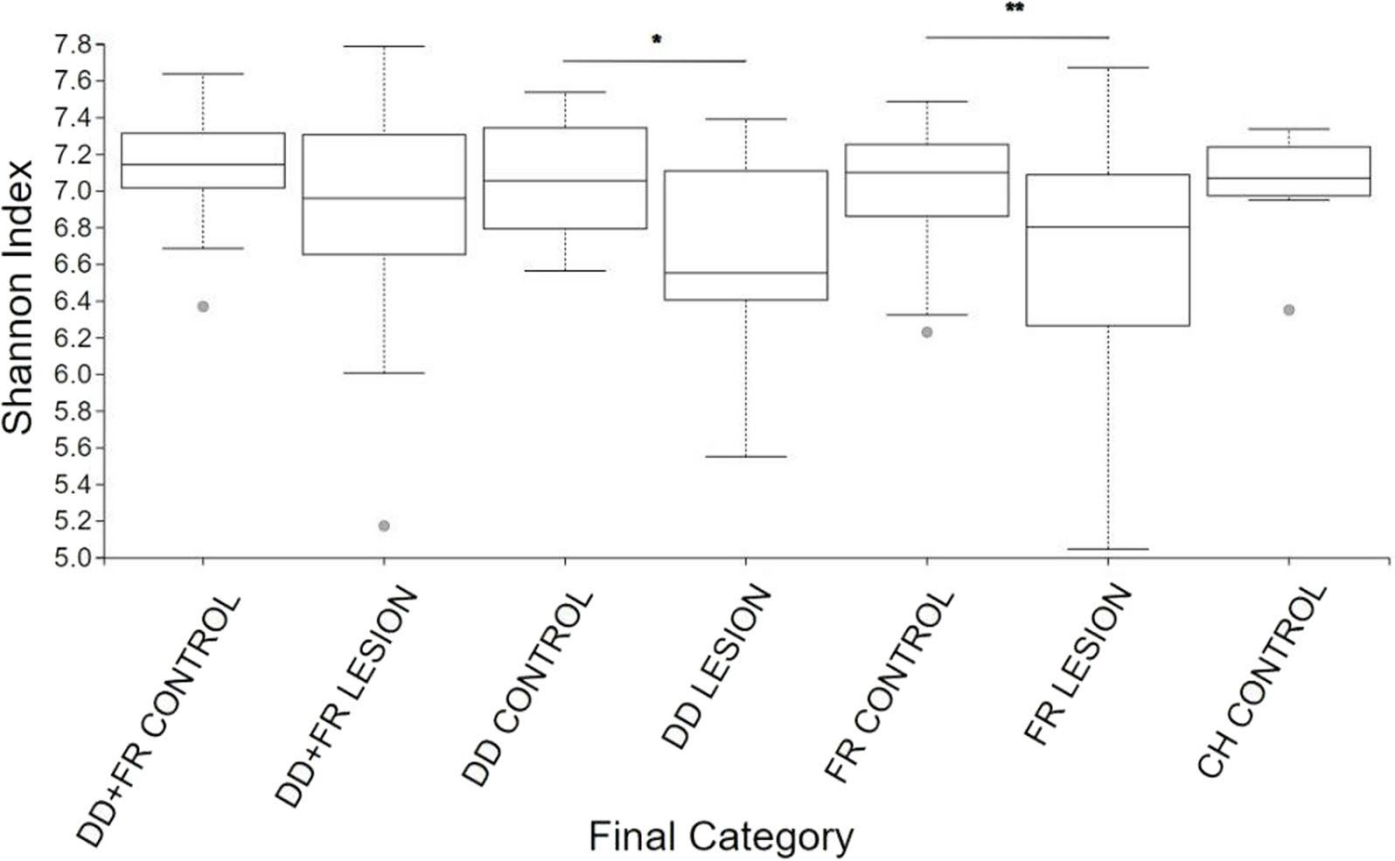
Shannon diversity index of bacterial communities of DD + FR control, DD + FR lesion, DD control, DD lesion, FR control, FR lesion and CH control. Pairwise analysis compares Shannon index of lesion and control skin microbial communities within a lesion type. *indicates an adjusted *p* value between 0.01 and 0.05, **indicates an adjusted *p* value < 0.01. DD = Digital dermatitis, FR = Foot rot, DD + FR = DD and FR co-infection in the same foot, CH control = control skin from completely healthy animals without any hoof lesions

DD and FR bacterial communities were less diverse (adj-*P* < 0.05) compared to their respective healthy skin communities, as indicated by Shannon and Chao1 indices (Figs. 2 and 3). However, the bacterial diversity of DD + FR lesions was not different from DD + FR controls. When the diversity of contralateral control skin in animals diagnosed with hoof lesions (DD, DD + FR, FR control) was compared to skin bacterial community of completely healthy cattle (CH control), there were no significant differences in either alpha diversity indices.

**Fig. 4:**
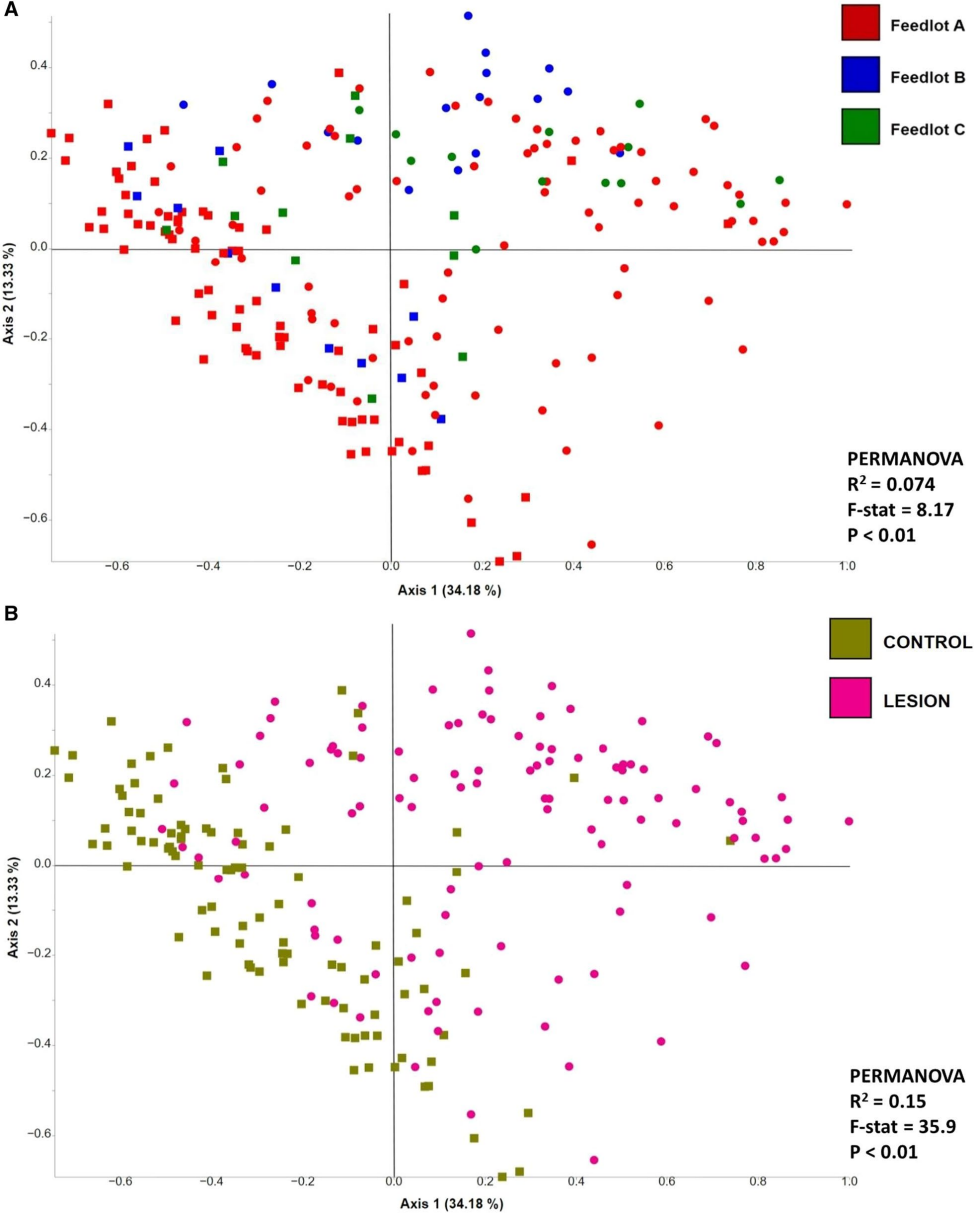

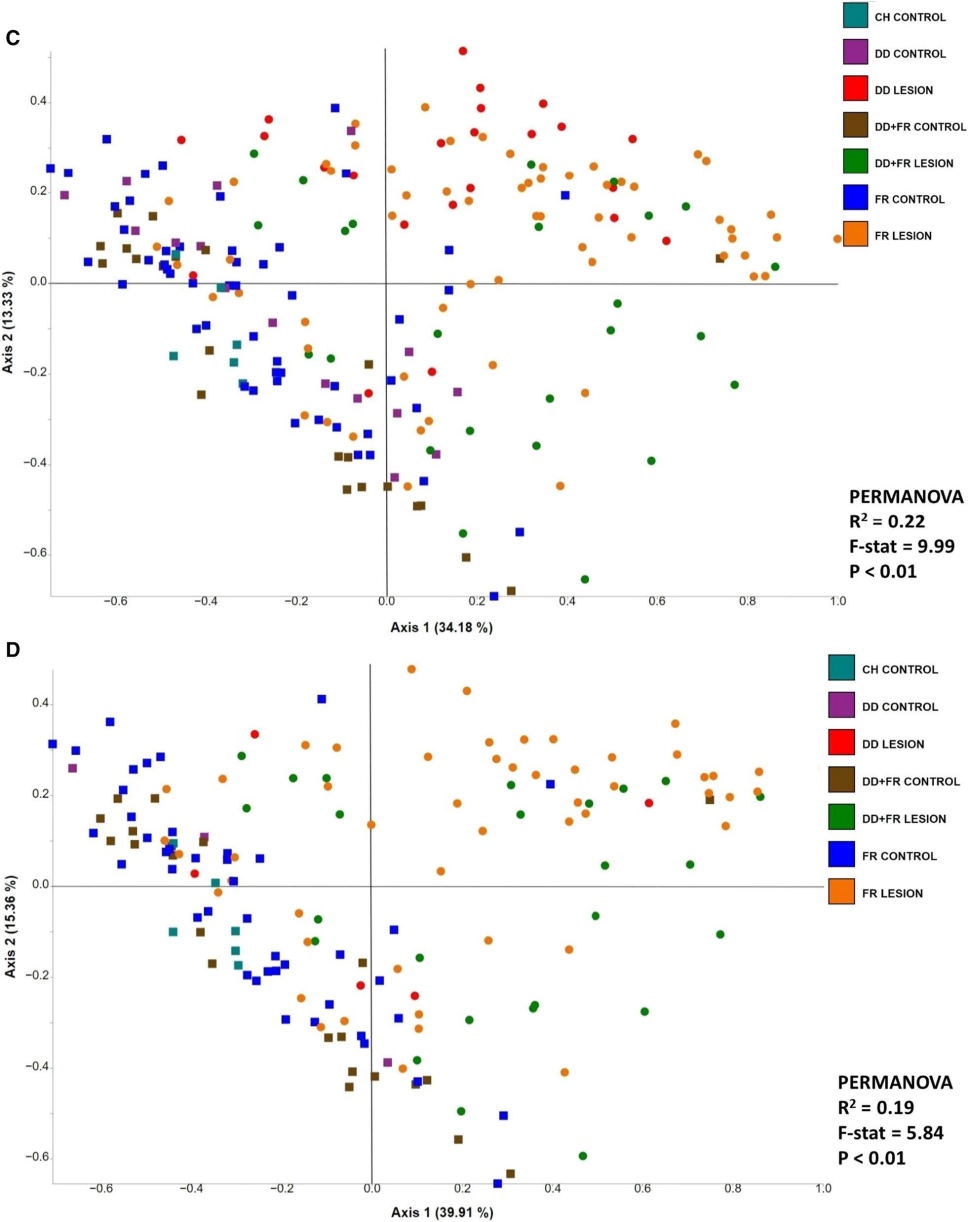

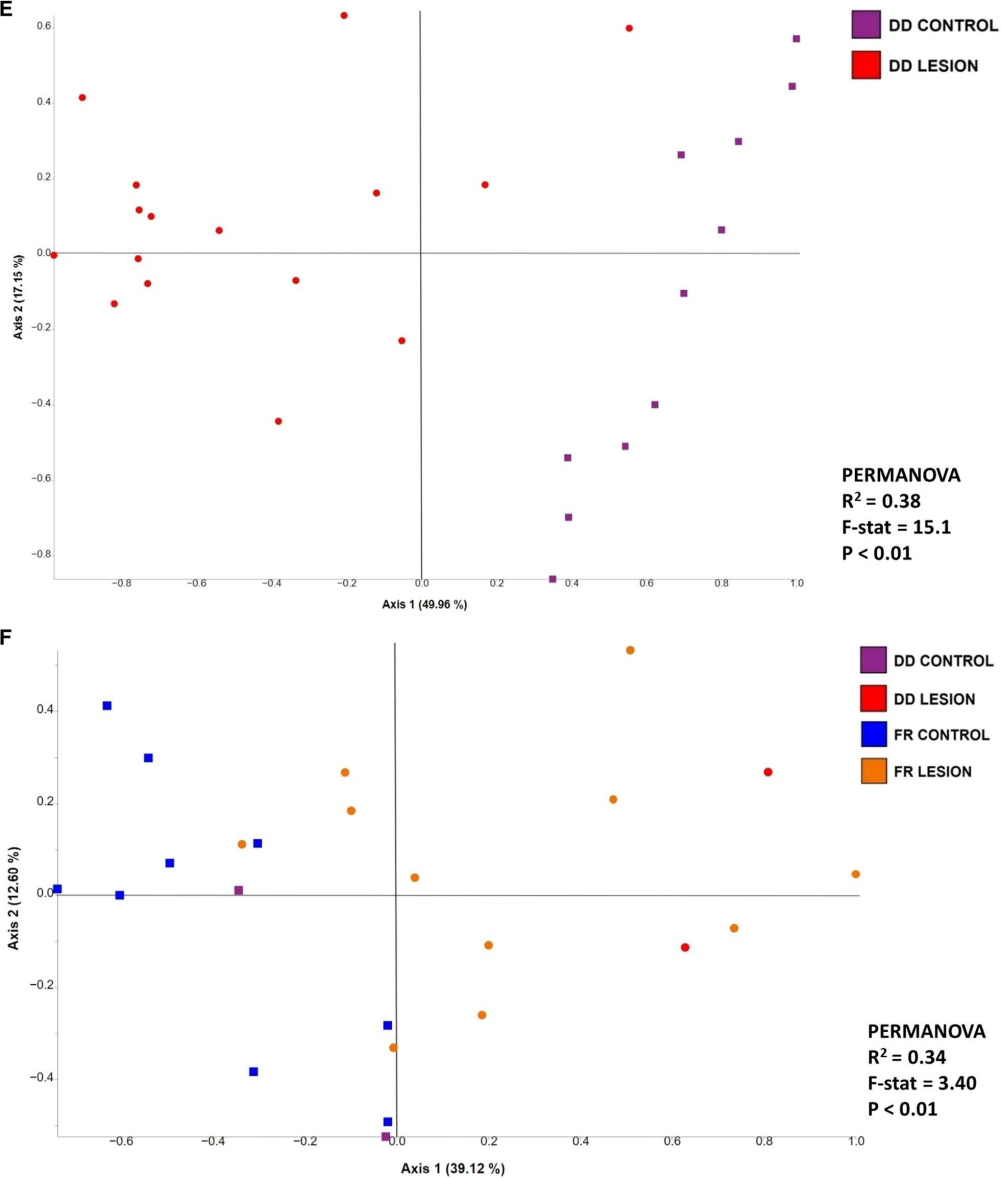
Principle coordinate analysis plot of weighted Unifrac distance metric generated from hoof bacterial communities showing **A** the effect of feedlots on bacterial communities,** B** the effect of sample type (lesion vs. control) on bacterial communities, **C** the effect of lesion type on bacterial communities, **D** the effect of lesion type on bacterial communities within feedlot A, **E** the effect of lesion type on bacterial communities within feedlot B, and **F** The effect of lesion type on bacterial communities within feedlot C. Squares represent control samples and circles lesion samples. DD = Digital dermatitis, FR = Foot rot, DD + FR = DD and FR co-infection in the same foot, CH control = control skin from completely healthy animals without any hoof lesions

Feedlot accounted for 7.4% of the observed variation of the beta diversity of bacterial communities (PERMANOVA-R^2^ = 0.074, F = 8.17, *P* < 0.01; Fig. 4A). In contrast, bacterial communities generated from all control skin samples (DD control, DD + FR control, FR control, and CH control) were clustered apart from lesion samples, regardless of the lesion type (Fig. 4B). Moreover, the sample type (lesion versus control skin) accounted for 15% of the observed variation between bacterial communities (PERMANOVA-R^2^ = 0.15, F = 35.9, *P* < 0.01). When the bacterial communities generated from lesion types and their respective control skin of all three feedlots were compared, lesion type contributed to 22% of the variation observed among bacterial communities (PERMANOVA-R^2^ = 0.22, F = 9.99, *P* < 0.01; Fig. 4C). *Post-hoc* pairwise comparisons revealed that bacterial profiles generated from all three lesion types were different (*P* < 0.05) from their corresponding control groups (Additional file 1: Table S3). Similar clustering patterns were observed within each feedlot when comparing bacterial communities generated from various lesion types against their respective control skin (Fig. 4D–F).

### Differential abundance analysis

Overall, there were 16 DA bacterial taxa between the lesion and control skin across each lesion type comparison, with some taxa associated with more than one type of lesion (Table 2). Specifically, there were 11, 12, and 3 DA taxa in DD, DD + FR, and FR-affected cattle, respectively. Of the 12 DA taxa found between DD + FR lesion and DD + FR control, relative abundance of 11 taxa were higher in DD + FR lesion compared to corresponding control skin. The relative abundance of only one taxon (*Lachnospiraceae_NK3A20_group*) was higher in DD + FR control compared to the DD + FR lesion (Table 2). In contrast, the relative abundance of DA taxa in DD and FR cattle was higher in the lesion compared to the corresponding control skin (Table 2). Songbird analysis revealed taxa associated with different lameness types (Additional file 1: Table S4). In the top 15 taxa associated with each lesion type, eight recurring taxa were associated with all three types of lesions, including *Fusobacterium, Lentimicrobium, Mycoplasma, Helcococcus, Fenollaria, [Eubacterium]_yurii_group, Succiniclasticum*, and *Porphyromonas*. Further overlapping taxa included: *Peptococcus* and *Peptostreptococcus* associated with DD and FR lesions; *Filifactor* and *Erysipelatoclostridium* associated with DD and DD + FR lesions; and *Trueperella* and *Parvimonas* associated with DD + FR and FR lesions.

**Table 2:** ALDEx2 output of differential abundant bacterial taxa in specific lesion type pairwise comparisons

Taxon	Lesion type	Control CLR	Lesion CLR	Effect size	Adj *P* value
Peptostreptococcaceae	DD	0.85	7.1	1.44	< 0.01
Mycoplasma	DD	− 1.53	6.94	1.24	< 0.01
Amnipila	DD	− 3.18	7.09	0.98	< 0.01
Acholeplasma	DD	− 0.64	5.7	0.94	< 0.01
Porphyromonas	DD	6.23	9.36	0.9	< 0.01
Murdochiella	DD	− 3.06	4.34	0.89	0.014
Fusobacterium	DD	− 2.84	4.59	0.87	< 0.01
Treponema	DD	4.93	9.82	0.87	0.032
Roseburia	DD	− 2.21	6.84	0.85	0.016
Campylobacter	DD	− 1.48	4.65	0.81	0.017
Filifactor	DD	− 3.59	0.98	0.8	0.013
Fusobacterium	DD + FR	− 2.43	8.71	2.12	< 0.01
Porphyromonas	DD + FR	3.85	9.53	1.32	< 0.01
Succiniclasticum	DD + FR	− 2.49	6.55	1.2	< 0.01
Peptostreptococcus	DD + FR	− 2.28	6.24	1.13	< 0.01
Peptoniphilus	DD + FR	2.95	7.43	1.07	< 0.01
Peptococcus	DD + FR	− 2.28	3.78	1.07	< 0.01
Helcococcus	DD + FR	1.26	6.07	0.99	< 0.01
Campylobacter	DD + FR	− 2.47	4.35	0.93	< 0.01
S5-A14a	DD + FR	− 3.2	3.48	0.85	0.011
Mycoplasma	DD + FR	− 3.14	5.37	0.84	< 0.01
[Eubacterium]_yurii_group	DD + FR	− 2.53	4.07	0.8	< 0.01
Lachnospiraceae_NK3A20_group	DD + FR	5.22	− 1.21	− 1.18	< 0.01
Fusobacterium	FR	− 1.45	9.45	1.05	< 0.01
Porphyromonas	FR	4.47	9.93	1.02	< 0.01
Peptostreptococcus	FR	− 1.96	7.04	0.9	< 0.01

CLR—median value of a genus’s transformed abundance data in the lesion or control skin group of samples; a higher CLR indicates a higher abundance

Adj *P* value—Benjamini–Hochberg adjusted *P* value controlled for false discovery rate

ALDEx2 transforms the compositional abundance data into centered log ratios (CLR) for taxa in the lesion and control skin group. It then compares them using a two-sample Welch’s t-test to determine significance. Only significant taxa with adj *P* value < 0.05 and the effect size > 0.8 are shown

### Taxonomic summary

**Fig. 5:**
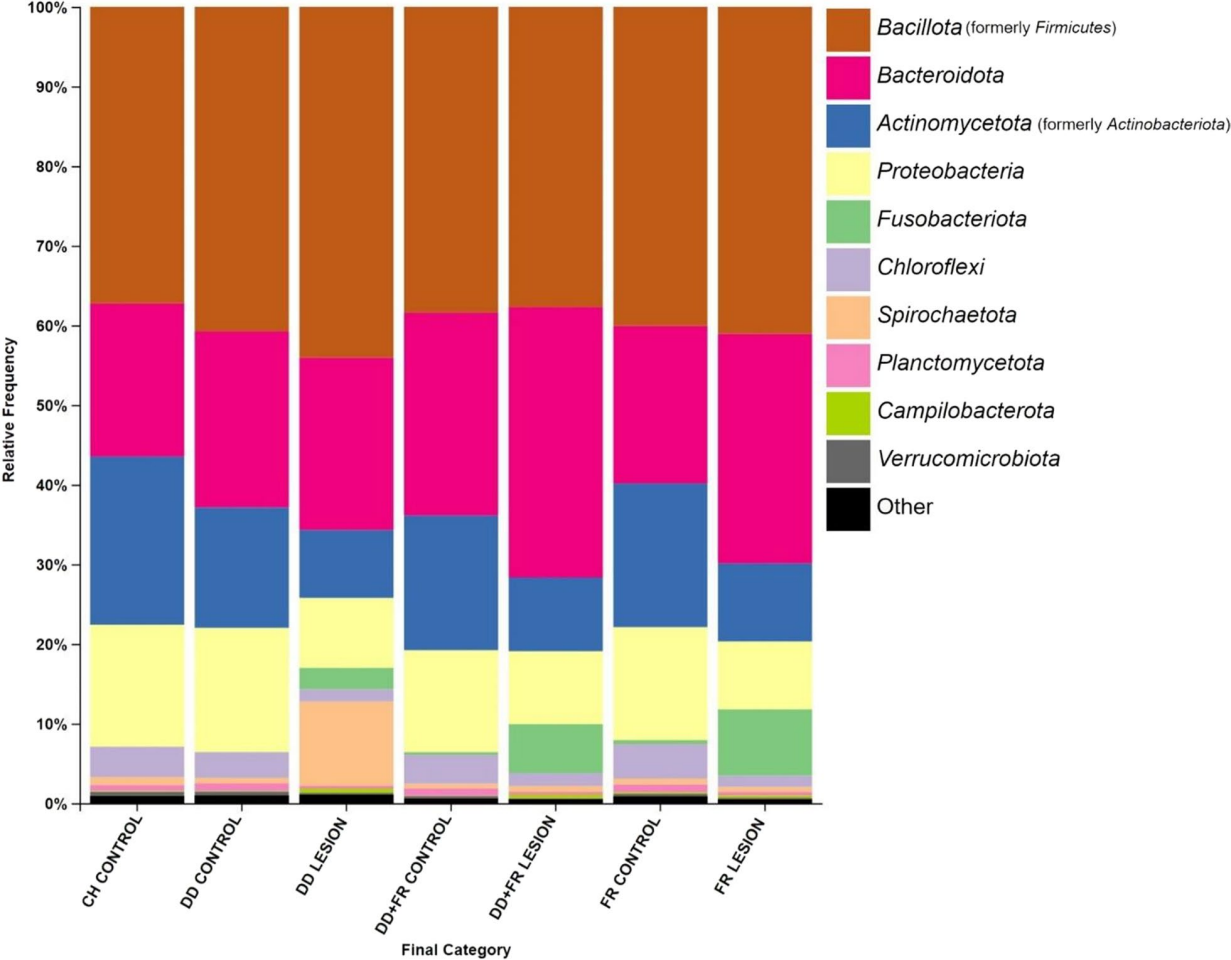
Taxonomic summaries of each hoof lesion or control skin category showing the percent relative abundance of the top 10 bacterial phyla

*Bacillota* (formerly *Firmicutes*), *Bacteroidota*, *Actinomycetota* (formerly *Actinobacteriota*), and *Proteobacteria* were the most prevalent phyla among all sample types (Fig. 5). DD lesion samples showed increased relative abundance of *Spirochaetota*, FR and DD + FR lesions showed increased relative abundance of *Fusobacteriota*, along with DD lesions but to a lesser extent (Fig. 5).

## Discussion

Surface swabs successfully revealed lesion-associated perturbations in hoof bacterial communities of feedlot cattle with common hoof lesions. The altered bacterial diversity of lesion categories indicated dysbiosis of surface bacterial communities [8]. The bacterial communities of lesions were distinct from the control bacterial communities, which were further marked by the increased abundance of several known pathogenic genera found in lesion categories. Pairwise comparisons between lesion and corresponding control skin revealed differences in weighted Unifrac distance of all three lesion types (Additional file 1: Table S3). The impact of feedlot was minimal, accounting for 7.4% of the differences in bacterial communities observed among samples, which was expected. In contrast, the sample type (whether it was a lesion or control sample) and the specific lesion/control category (DD lesion, FR lesion, DD + FR lesion, DD control, FR control, DD + FR control, or CH control) contributed 15% and 22%, respectively, to changes in bacterial communities. In general, these results were in accordance with our hypotheses that lesion communities are different from those of control skin communities and contain an abundance of pathogenic taxa in lesions. In a previous study, Bay et al. [8] reported similar beta diversity findings from hoof swabs obtained from various claw lesions, including FR, compared to control skin samples. Our findings are also consistent with previous biopsy-based DD studies that found significant differences in bacterial composition between DD lesions and control skin [16, 29–32]. Profiling the bacterial communities using 16S rRNA amplicon sequencing can provide an objective way to differentiate these two hoof lesions that can occur in proximity that may overlap with one another. Hence, there is potential for swabs to be used in laboratory diagnosis.

The results of this swab-based study are consistent with DD sequencing studies that utilized skin lesion biopsies, which also reaffirm our expectation that swabs are a viable sampling method. Although tissue biopsy has been the gold standard in DD lesion microbiome research, the technique is time and labour intensive as well as invasive, and may interfere with the healing process. These results are consistent with a previous study assessing DD in dairy cattle using lesion surface swabs and genus- or species-specific PCR to detect the presence or absence of common hoof pathogens such as *Treponema* [33]. However, in the present study, lesion surface swabs provided insights into the bacterial communities associated with both lesion and control skin through 16S rRNA amplicon sequencing. Not only did this sampling approach show a separation in community structures between lesion types (DD, FR, or DD + FR) and control skin within affected animals, but the differential abundance analysis also revealed an increased abundance of bacterial taxa commonly associated with DD and FR such as *Fusobacterium*, *Porphyromonas, Treponema*, *Mycoplasma* and *Campylobacter* [8, 16, 29]. Thus, surface swabbing, combined with 16S rRNA amplicon sequencing, showed to be a non-invasive method for describing bacterial communities of hoof lesions. Along with the declining costs of sequencing technology, cotton swabs can meaningfully lower the processing and cost threshold associated with hoof lesion research while enabling a larger number of samples to be analyzed.

Differential abundance analysis indicated that DD and FR involve multiple pathogens, which further supports the polymicrobial understanding of hoof lesions [17]. ALDEx2 is a compositionally aware DA tool that enables direct comparison between a type of lesion and its corresponding control, within animal (eg., DD lesion vs DD control). On the other hand, Songbird used a multinomial approach because it can account for feedlot differences which included the entire dataset (n = 208) in the analysis. Several taxa (*Fusobacterium, Porphyromonas, Mycoplasma, Campylobacter* and *Peptostreptococcus*) had increased abundance in more than one type of hoof lesion, indicating they may be associated with both DD and FR (Table 2). While DD + FR lesions were expected to resemble a combination of DD and FR, we did not expect to find similar groups of bacteria in DD and FR lesions. Involvement of traditionally FR-associated pathogens *Fusobacterium* and *Porphyromonas* is not novel [16, 30], as synergism between *F. necrophorum* and *P. levii* generates a mixed species biofilm that can impair the immune response of the bovine host [34, 35]. However, the Songbird analysis suggests more bacterial groups may be involved across both DD and FR lesions. This is because after accounting for the feedlot effect, the same taxa remained highly associated with each lesion and consistently appeared within the top 15 taxa of each lesion type (Additional file 1: Table S4). While there is substantial evidence that *Treponema* is clearly associated with DD [19], *Peptostreoptococus, Helcococcus*, *Fusobacterium* and *Porphyromonas* have all been associated with both DD and FR lesions [8, 16, 29], suggesting a potential aetiological connection.

There are several limitations related to the sampling and processing in this study. Some inherent to amplicon sequencing include potential biases during the PCR due to the selection of the hypervariable region. The relatively short read of the V4 hypervariable region (250 base pairs) did not provide reliable species-level identification but at the genus level. Fecal contamination was also a potential issue with surface swabbing; however, the filtering procedure and inclusion of a within-animal control mitigated this issue. The sample size was also a potential concern as there were more samples in the FR categories (FR lesion and FR control) than other types of samples and only five healthy negative control samples. Furthermore, the cross-sectional design of this study only captured the bacterial communities of lesions at a single point in time, wherein the majority of the DD cases were either at the M2 or M4.1 stage of their lesion (Additional file 1: Table S5). Unlike in DD lesions, *Treponema* was not a DA genus in DD + FR lesions. More samples to include DD + FR might be able to capture alterations in the hoof bacterial community when both lesion types co-exist. Moreover, future studies that swab DD and FR lesions separately (when they co-exist) will provide an in-depth understanding of microbial ecology of this particular type of lesion; there should be a focus on distinct lesions of the hoof whilst avoiding areas where DD and FR intersect or in close proximity with each other.

## Conclusion

In summary, this is the first study to describe the surface community of a DD and FR combination lesions in beef cattle using 16S rRNA sequencing. The bacterial communities of hooves affected with DD, FR, or DD + FR were significantly different from the corresponding healthy skin taken from the same animal. The lesion or control hoof samples (Final Category) was the single most important driver for changes in bacterial communities. The lesion-specific effect remained when analyzed within samples from the same feedlot. Surface swabbing the lesion appears to be a functional, non-invasive alternative to lesion biopsy in hoof lesion research. Under feedlot conditions where a visual diagnosis can be difficult, cotton swabs can be used to develop a rapid chute side test that targets key taxa such as *Treponema*, *Fusobacterium* and *Porphyromonas* for infectious lameness detection. Several bacterial genera had increased abundance in DD, FR, and DD + FR lesions, indicating there is overlap in some pathogens associated with both DD and FR. Compared to control skin, *Fusobacterium* and *Porphyromonas* had increased abundance in all three types of lesions studied. Future studies should investigate a possible shared etiology between these two hoof diseases, largely considered to be unrelated. These bacterial results build on the polymicrobial view of not only DD but also provided the bacterial characterization of FR in beef cattle through a practical and non-invasive method of sampling.

### Supplementary Information

**Additional file 1: Table S1.** Biological information of cattle sampled in this study. Health status refers to whether an animal has hoof disease/lesions. Healthy animals without any hoof lesions (which provided CH control samples) were from feedlot A. **Table S3.** Beta diversity analysis of PERMANOVA pairwise comparison between lesion type and control skin groups (final categories) based on weighted Unifrac distance metric. **Table S4.** The top 15 associated taxa with each hoof lesion type in Songbird analysis. Single-underlined taxa were associated with at least two lesion types, and double-underlined taxa were associated with all three lesion types. **Table S5.** Distribution of different M-stages of lesions in DD-lesion and DD+FR lesion samples, using the Dopfer (1997) M-stage 5-point classification system across three feedlots. M-stage only applies to the DD lesion in the DD+FR lesions. No M3 and only one M1 lesion were observed in trial period.

**Additional file 2: Table S2.** Samples sequence quality test reults.

## Data Availability

Sequence data were deposited at NCBI Sequence Read Archive (SRA) under accession number PRJNA940284.

## Abbreviations

DD: Digital dermatitis
FR: Foot rot
DD + FR: Digital dermatitis and foot rot co-infection
DA: Differential abundance
PCR: Polymerase chain reaction
ASV: Amplicon sequence variants
PERMANOVA: Permutational multivariate analysis of variance
ALDEx2: ANOVA-Like differential gene expression analysis 2
PCoA: Principal coordinates analysis
rRNA: Ribosomal ribonucleic acid
CH control: Completely healthy control
QIIME2: Quantitative insights into microbial ecology 2
DADA2: Divisive amplicon denoising algorithm 2
