# Supplementary material for: Characterization of the hoof bacterial communities in feedlot cattle affected with digital dermatitis, foot rot or both using a surface swab technique
Source: Anim Microbiome. 2024 Jan 22;6:2. doi: 10.1186/s42523-023-00277-1 (PMC10804539; doi:10.1186/s42523-023-00277-1)
Supplement: Supplementary file 1 — Additional file 1: Table S1. Biological information of cattle sampled in this study. Health status refers to whether an animal has hoof disease/lesions. Healthy animals without any hoof lesions (which provided CH control samples) were from feedlot A. Table S3. Beta diversity analysis of PERMANOVA pairwise comparison between lesion type and control skin groups (final categories) based on weighted Unifrac distance metric. Table S4. The top 15 associated taxa with each hoof lesion type in Songbird analysis. Single-underlined taxa were associated with at least two lesion types, and double-underlined taxa were associated with all three lesion types. Table S5. Distribution of different M-stages of lesions in DD-lesion and DD+FR lesion samples, using the Dopfer (1997) M-stage 5-point classification system across three feedlots. M-stage only applies to the DD lesion in the DD+FR lesions. No M3 and only one M1 lesion were observed in trial period. [file 42523_2023_277_MOESM1_ESM.docx]

| Feedlot | Health status | Heifers (n) | Steers (n) | Total (n) | Average weight (lb) | Average temperature (F°) | Average locomotion score | Average sampling date |
| --- | --- | --- | --- | --- | --- | --- | --- | --- |
| Feedlot A | Healthy | 2 | 3 | 5 | 1150.0 | 103.8 | 0.2 | 2019-06-28 |
|  | Disease | 34 | 39 | 74 | 1011.1 | 104.0 | 1.8 | 2019-07-01 |
| Feedlot B | Disease | - | 11 | 11 | 1358.5 | 102.8 | 1.7 | 2020-03-08 |
| Feedlot C | Disease | 11 | - | 11 | 1353.0 | 102.2 | 2.4 | 2020-03-09 |
|  | Total | 47 | 53 | 101 | 1093.9 | 103.7 | 1.8 | 2019-08-24 |

Table S1. Biological information of cattle sampled in this study. Health status refers to whether an animal had hoof lesions indicative of disease. Healthy control animals without any hoof lesions (CH control samples) originated from Feedlot A.

Table S3. Beta diversity analysis of PERMANOVA pairwise comparison between lesion type and control skin groups based on weighted Unifrac distance metric.

| **Pairwise Comparison** | | **PERMANOVA** | | | |
| --- | --- | --- | --- | --- | --- |
| **Group 1** | **Group 2** | **Sample size** | **F-stat** | **R^2^** | **Adj *P*-value** |
| DD+FR lesion | DD+FR control | 47 | 8.66 | 0.16 | <0.01 |
|  | DD lesion | 48 | 10.26 | 0.18 | <0.01 |
|  | FR lesion | 87 | 1.86 | 0.02 | 0.10 |
|  | CH control | 31 | 6.75 | 0.19 | <0.01 |
| DD lesion | DD control | 39 | 10.54 | 0.22 | <0.01 |
|  | FR lesion | 85 | 10.16 | 0.11 | <0.01 |
|  | CH control | 29 | 8.12 | 0.23 | <0.01 |
| FR lesion | FR control | 114 | 22.20 | 0.17 | <0.01 |
|  | CH control | 68 | 6.21 | 0.09 | <0.01 |

Adj *P*-value - Benjamini-Hochberg adjusted *P*-value controlled for false discovery rate. Significance at 0.05.

Table S4. Songbird analysis showing the top 15 taxa associated with each type of hoof lesion. Single-underlined taxa were associated with at least two lesion types, and double-underlined taxa were associated with all three lesion types.

| **Rank** | **DD associated taxon** | **DD+FR associated taxon** | **FR associated taxon** |
| --- | --- | --- | --- |
| 1 | *^§^Lentimicrobium* | *^¶^Fusobacterium* | *^¶^Fusobacterium* |
| 2 | *^¶^Fusobacterium* | *^†^[Eubacterium]_yurii_group* | *^†^Helcococcus* |
| 3 | *^†^Mycoplasma* | *^§^Lentimicrobium* | *^†^Peptococcus* |
| 4 | *^†^Helcococcus* | *^†^Mycoplasma* | *^†^Peptostreptococcus* |
| 5 | *^†^Veillonella* | *^†^Fenollaria* | *^*^Trueperella* |
| 6 | *^†^Fenollaria* | *^*^Trueperella* | *^†^S5-A14a* |
| 7 | *^†^Amnipila* | *^†^Filifactor* | *^§^Porphyromonas* |
| 8 | *^†^Peptococcus* | *^§^Porphyromonas* | *^†^Succiniclasticum* |
| 9 | *^†^Filifactor* | *^†^Helcococcus* | *^§^Lentimicrobium* |
| 10 | *^†^Erysipelatoclostridium* | *^†^Parvimonas* | *^†^[Eubacterium]_yurii_group* |
| 11 | *^†^[Eubacterium]_yurii_group* | *^†^Peptococcus* | *^†^Mycoplasma* |
| 12 | *^†^Peptostreptococcus* | *^†^Peptostreptococcaceae* | *^†^Parvimonas* |
| 13 | *^†^Catonella* | *Dichelobacter* | *^†^Murdochiella* |
| 14 | *^†^Succiniclasticum* | *^†^Erysipelatoclostridium* | *^†^Fenollaria* |
| 15 | *^§^Porphyromonas* | *^†^Succiniclasticum* | *^§^Proteiniphilum* |

| Phylum |  |
| --- | --- |
| ****Actinomycetota (*formerly *Actinobacteriota)* | |
| *†**Bacillota (*formerly *Firmicutes)* | |
| *§Bacteroidota* | |
| *¶Fusobacteriota* | |

|  | CH control | DD control | DD lesion | | | | DD+FR control | DD+FR lesion | | |
| --- | --- | --- | --- | --- | --- | --- | --- | --- | --- | --- |
| M-Stage | **M0** | **M0** | **M1** | **M2** | **M4** | **M4.1** | **M0** | **M2** | **M4** | **M4.1** |
| Feedlot A | 6 | 3 | 1 | 1 | 2 | 1 | 22 | 11 | 3 | 11 |
| Feedlot B | 0 | 11 | 0 | 4 | 0 | 12 | 0 | 0 | 0 | 0 |
| Feedlot C | 0 | 2 | 0 | 2 | 0 | 0 | 0 | 0 | 0 | 0 |
| Total | 6 | 16 | 1 | 7 | 2 | 13 | 22 | 11 | 3 | 11 |

Table S5. Distribution of the DD lesions using the Dopfer (1997) 5-point, M-stage classification system. No M3 and only one M1 lesion were observed.

DD - Digital dermatitis

FR- Foot rot

DD+FR- DD and FR co-infection in the same foot

CH control - animals without any hoof lesions

CH control, DD+FR lesion and DD+FR control samples came only from feedlot A.

Feedlot B contributed DD lesion and DD control samples only.
